# Cardiovascular Risk After SARS-CoV-2 Infection Is Mediated by IL18/IL18R1/HIF-1 Signaling Pathway Axis

**DOI:** 10.3389/fimmu.2021.780804

**Published:** 2022-01-05

**Authors:** Liwei Zhang, Mingxing Li, Zhiwei Wang, Peng Sun, Shunbo Wei, Cong Zhang, Haoliang Wu, Hualong Bai

**Affiliations:** ^1^ Department of Vascular and Endovascular Surgery, First Affiliated Hospital of Zhengzhou University, Zhengzhou, China; ^2^ Key Vascular Physiology and Applied Research Laboratory of Zhengzhou City, Zhengzhou, China

**Keywords:** COVID-19, cardiovascular risk, IL18, IL18R1, HIF-1 signaling pathway

## Abstract

**Objectives:**

Currently, cardiovascular risk associated with COVID-19 has been brought to people’s attention, but the mechanism is not clear. The aim of this study is to elucidate the mechanisms based on multiple omics data.

**Methodology:**

Weighted gene co-expression network analysis (WGCNA) was used to identify key pathways. Combination analysis with aneurysm and atherosclerosis related pathways, hypoxia induced factor-1 (HIF-1) signaling were identified as key pathways of the increased cardiovascular risk associated with COVID-19. ScMLnet algorithm based on scRNA-seq was used to explore the regulation of HIF-1 pathway by intercellular communication. Proteomic analysis was used to detect the regulatory mechanisms between IL18 and HIF-1 signaling pathway. Pseudo time locus analysis was used to study the regulation of HIF1 signaling pathway in macrophages and vascular smooth muscle cells (VSMC) phenotypic transformation. The Virtual Inference of protein-activity by Enriched Regulon (VIPER) analysis was used to study the activity of regulatory proteins. Epigenetic analysis based on methylation revealed epigenetic changes in PBMC after SARS-CoV-2 infection. Potential therapeutic compounds were explored by using Cmap algorithm.

**Results:**

HIF-1 signaling pathway is a common key pathway for aneurysms, atherosclerosis and SARS-CoV-2 infection. Intercellular communication analysis showed that macrophage-derived interleukin-18 (IL-18) activates the HIF-1 signaling pathway through IL18R1. Proteomic analysis showed that IL18/IL18R1 promote NF-κB entry into the nucleus, and activated the HIF-1 signaling pathway. Macrophage-derived IL18 promoted the M1 polarization of macrophages and the syntactic phenotype transformation of VSMCs. MAP2K1 mediates the functional regulation of HIF-1 signaling pathway in various cell types. Epigenetic changes in PBMC after COVID-19 infection are characterized by activation of the type I interferon pathway. MEK inhibitors are the promising compounds for the treatment of HIF-1 overactivation.

**Conclusions:**

The IL18/IL18R1/HIF1A axis is expected to be an therapeutic target for cardiovascular protection after SARS-CoV-2 infection. MEK inhibitors may be an choice for cardiovascular protection after SARS-COV-2 infection

## Introduction

COVID-19 is a global respiratory epidemic caused by SARS-CoV-2, currently infects at least 68 million people, and the number of cases is rising with the emergence of mutated strains such as Delta (1) and Omicron (2). After SARS-CoV-2 infection, approximately 20% of patients will develop moderate to severe symptoms, manifested as acute respiratory distress syndrome (ARDS), with elevated plasma proinflammatory factors (3). At present, clinical studies have shown that patients with other diseases such as hypertension, diabetes, cardiovascular disease and respiratory disease have a poor prognosis and a higher mortality rate (4). In terms of gender, male patients have a higher mortality and more severe symptoms rate than female patients (5). In addition, age is also an independent risk factor affecting the prognosis of patients with COVID-19, and elderly patients have a poor prognosis.

Clinical studies have shown that the severity of COVID-19 is positively correlated with levels of inflammatory factors (6, 7), which can cause ARDS, diffuse intravascular coagulation and multiple organ failure. Induction of these cytokines is mediated by (8): (I) the angiotensin II/AT1R pathway; (ii) ACE2 signaling pathway; (iii) Innate immune signaling pathways, including PRRs such as TLRs, RIG-1, and MDA5 signaling pathways, nucleotide binding oligomer domains (NOD), Leucine-rich repeat domain (LRR), and Pyrin domain-containing protein 3 (NLRP3) inflammasome. In addition, the HIF-1 signaling pathway participate in the process of SARS-CoV-2 infection (9) and aggravate the inflammatory response to COVID-19 (10).

Clinical symptoms of COVID-19 are highly heterogeneous, ranging from asymptomatic infection to ARDS (11). Multiple organs involvement, including the cardiovascular system, have been observed in severe patients. Recent meta-analysis results suggest that cardiovascular diseases (CVD) and its risk factors (hypertension and diabetes) are strongly associated with mortality rate in COVID-19 patients of all ages (12). Current autopsies of COVID-19 patients have showed evidence of viral particles within vascular endothelial cells in the cardiovascular system and diffuse vascular endothelial cell damage (13). Inflammatory response to viral infection up-regulates tissue factor expression, thrombin production, platelet and complement activation; thus increased the risk of intravascular thrombosis (14). A wide range of cardiovascular sequelae, including acute onset heart failure, arrhythmias, acute coronary syndrome, myocarditis, and cardiac arrest, appear to be associated with COVID-19 (15). However, COVID-19 associated risks of peripheral vascular diseases such as aneurysms and atherosclerosis have not been studied.

In this study, we explored key signaling pathways based on RNA-seq data of SARS-CoV-2 infection, aneurysms and atherosclerosis. The IL18/IL18R1/HIF-1 signaling axis was found to mediate an increased risk of peripheral vascular disease such as aneurysms and atherosclerosis after COVID-19. Epigenetic changes in immune cells after SARS-CoV-2 infection promotes the expression of IL18. We found that MAP2K1 plays a key role and that MEK inhibitors can be used as treatment options for cardiovascular sequelae after COVID-19.

## Materials and Methods

### Data Acquisition

Data sets GSE120521, GSE156754, GSE7084, GSE57691, GSE98278, GSE157859, GSE152418, GSE171668, and GSE174818, derived from GEO database. Each dataset was designed as follows:

**GSE120521**: Atherosclerotic plaques were obtained from 4 patients and dissected in stable and unstable areas.

**GSE156754**: Pluripotent stem cells (iPSCs) were induced into cardiomyocytes, cardiac fibroblasts and endothelial cells, and the transcriptome changes were observed 48 hours after SARS-CoV-2 infection and simulated infection.

**GSE7084**: Abdominal aorta (7 samples) and abdominal aortic aneurysm (6 samples) samples were obtained at autopsy and surgically, respectively, and transcriptome sequencing was performed to explore differences in expression.

**GSE57691**: Genome-wide expression analysis of abdominal aortic aneurysm (AAA) and aortic occlusive disease (AOD) specimens obtained from 20 patients with small AAA (mean maximum aortic diameter=54.3 ± 2.3 mm), 29 patients with large AAA (mean maximum aortic diameter=68.4 ± 14.3 mm), and 9 AOD patients (mean maximum aortic diameter=19.6 ± 2.6 mm). Relative aortic gene expression was compared with that of 10 control aortic specimen of organ donors.

**GSE98278**: The data set compares transcriptome of elective stable (n = 31) vs ruptured (n = 17) AAA and intermediate size (n = 15) vs large (n = 16) AAA.

**GSE157859**: A total of 18 COVID-19 patients were included, including 6 mild cases, 7 moderate cases and 5 severe cases. PBMC samples from each sample were collected at the treatment stage, convalescence stage, and rehabilitation stage, respectively, and RNA sequencing was performed.

**GSE152418**: RNAseq of PBMCs in a group of 17 COVID-19 subjects and 17 healthy controls.

**GSE171668**: The dataset contained 420 autopsy samples from 17 COVID-19 patients, covering 11 organs. In this study, single-cell sequencing data of heart samples from patients with COVID-19 were included.

**GSE174818**: Using Illumina HumanMethylation EPIC, methylation sequencing was performed on whole blood tissues from 102 patients and 19 non-COVID-19 patients to detect epigenetic changes in PBMC after COVID-19.

Proteomic analysis and matching of therapeutic potential compounds were performed by http://cpdb.molgen.mpg.de/CPDB and https://clue.io/query. The rest of the data processing was supported by R (4.0.5) and Python (3.9.5).

### Exploration of the Common Key Pathways in COVID-19, Aneurysms and Atherosclerosis

Weighted gene co-expression network analysis (WGCNA) explores regulatory modules through gene expression pattern and the correlation with relevant reference factors. Cardiomyocytes, endothelial cells, and fibroblasts in GSE156754 were induced by pluripotent stem cells. Unlike the original design of the dataset, this study does not consider the amount of virus used during infection. To identify specific transcriptome changes caused by SARS-CoV-2 infection, we performed WGCNA. By default, the soft threshold for network segmentation is calculated automatically. After mapping modules and groups, modules with positive correlation and P value less than 0.05 with COVID-19 infection group were selected for subsequent analysis. We did not consider the heterogeneity of cell types except for differentiation-related pathways. GSE7084, GSE57691 and GSE120521 were used to explore the key differential genes in aneurysm and atherosclerotic plaque, respectively. The results were shown in the volcano map. Only the upregulated genes were included in this study. (logFC>0.5 & adjust P value<0.1). Through the enrichment analysis, the key pathways of COVID-19, aneurysms and atherosclerosis were obtained. In the enrichment results, we focus only on signal transduction pathways.

### Analysis of Intercellular Communication Based on scMLnet Algorithm

ScMLnet is an R packet supported by Python for constructing intercellular/intracellular multilayer signal networks based on single-cell RNA-seq expression data. Based on specific gene expression, prior network information and statistical inference, scMLnet integrates intercellular pathways (ligand-receptor interactions) and intracellular subnetworks (receptor-TF pathways and TF-target gene interactions) to construct a multi-layer network. ScMLnet was implemented by using R (version 4.0.5) and Python (version 3.9.5).

Due to SARS-CoV-2 infection, macrophages infiltrated in the cardiovascular system. In this study, macrophages were used as the ligand, and cardiac myocytes, endothelial cells and vascular smooth muscle cells were used as the receptor cells. After combined analysis with aneurysms and atherosclerosis transcriptome differences, key genes were identified and labeled. We focused on the regulation mechanism related to HIF-1 signaling pathway and obtained the IL18/IL18R1/HIF-1 signaling pathway axis. The expression distribution of IL18 IL18R1 and HIF1A were plotted to demonstrate the source and the scope of regulation.

### Proteomics Analysis Based on CPDB

Consensuspathdb-human integrates the interaction network of Homo sapiens, including complex protein-protein, genetic, metabolic, signaling, gene regulation and drug target interactions, and biochemical pathways. Data were collected from 30 publicly available databases and interrelationships collated from the literature. Interactive data is integrated in complementary ways to form a seamless network of interactions involving different types of interactions. In this study, IL18R1 was used as the starting point of regulation and HIF1A was used as the endpoint of regulation. We only focused on direct interaction relationships, and all indirect regulatory relationships were filtered out.

### Related Pathway Expression Scores in the Samples Were Calculated Based on GSVA

Gene set variation analysis (GSVA) is a nonparametric, unsupervised method used to estimate the degree of enrichment of relevant gene sets from expression data. In this study, we evaluated the expressions of NF-κB and HIF-1 signaling pathway in each sample derived from GSE57691 and GSE98278 by GSVA score. The genes contained in NF-κB and HIF-1 signaling pathway are provided by R program package KEGGREST.

### The Expression of the Pathways Was Assessed by GSEA

Gene Set Enrichment Analysis (GSEA) is a computational method that determines whether an *a priori* defined set of genes shows statistically significant, concordant differences between two biological states. In this study, we divided the samples into high and low expression groups based on the expression level of IL18 in RNA-seq at bluk level for GSEA analysis to evaluate the expression of NF-κB and HIF-1 signaling pathway (GSE57691 and GSE98278).

### Single Cell Transcriptome Analysis

We performed single-cell transcriptome analysis based on GSE171668.The data were filtered and standardized, and then clustered to explore cell subtypes and determine the characteristic expression genes among clusters. It is worth noting that the annotations of cells in this study follow the annotations of the original data set. Because the cell annotation of the original data set has been manually corrected, it had high reliability. UMAP plot showed that the clustering results of this study were consistent with the original data. To investigate the regulation of the IL18/IL18R1/HIF-1 signaling pathway on cell phenotypes, we performed a pseudo-time locus analysis. Macrophages were annotated as M1 and M2 polarization directions, while smooth muscle cells were divided into contraction and synthetic phenotypic transformation directions. CD163 was the marker of M2 polarization, but it was noteworthy that the expression of traditional M1 macrophage markers(iNOS,CD86,TNFα) were low in the original data. Studies have shown that NAMPT promotes inflammation and M1 polarization of macrophages (16). Therefore, NAMPT was used as an indicator of M1 polarization in this study. ACTA1 and DES were used as contraction phenotype markers in smooth muscles, while MGP was used as synthetic phenotype markers.

### Virtual Inference of Protein-Activity by Enriched Regulon Analysis (VIPER)

At present, studies on the mechanism mainly rely on RNA-seq, which represents the functional state of the corresponding biological process through the expression of mRNA. However, mRNA expression does not constitute a reliable predictor of protein activity because it does not capture the various post-transcriptional and post-translational events. Proteomics based on mass spectrometry technology can better directly detect protein abundance, but still cannot directly obtain the active status of proteins. The VIPER algorithm allows computational inference of protein activity by using the expression of genes that are most directly regulated by a given protein. Regulatory network described in Human breast Carcinoma Network was used in this study (http://dx.doi.org/10.6084/m9.figshare.695962).

### Methylation Analysis Based on EPIC Chip

The methylation analysis in this study was performed by R package ChAMP based on Illumina 850K. GSE174818 included 102 patients with COVID and 26 patients with non-COVID. After quality control and probe filtration, differential methylation points (DMP) and differential methylation region (DMR) were explored. DMP of logFC<0 and pvalue<0.05 is considered as the core difference site. The core DMP locus was enriched and analyzed to detect epigenetic changes of PBMC after SARS-CoV-2 infection. In this study, we only focused on signaling pathways, and other biological processes and compounds were filtered out.

### Prediction of Potential Therapeutic Compounds

The Connectivity Map team at the Broad Institute has generated expression profiles from the gains and losses of thousands of chemical or genetic functional disruptors in a variety of cellular environments. A subset of these interferers is drugs, probes, or tool compounds with known activities and targets that have been well annotated in previous scientific literature. These perturbators have been systematically analyzed by CMap in nine cell lines. TAS is an aggregate measure of signature strength (SS) and replicate correlation (CC) which is intended to represent a perturbagen’s transcriptional activity. The more transcriptionally active a perturbagen, the higher its TAS. The CS score represents the similarity between the compound’s effect on the transcriptome and the reference transcriptome changes. In this study, we considered both the transcriptional activity score and CS score, and the total score was defined as the product of the two scores.

## Results

### Data Processing

**Figure 1:**
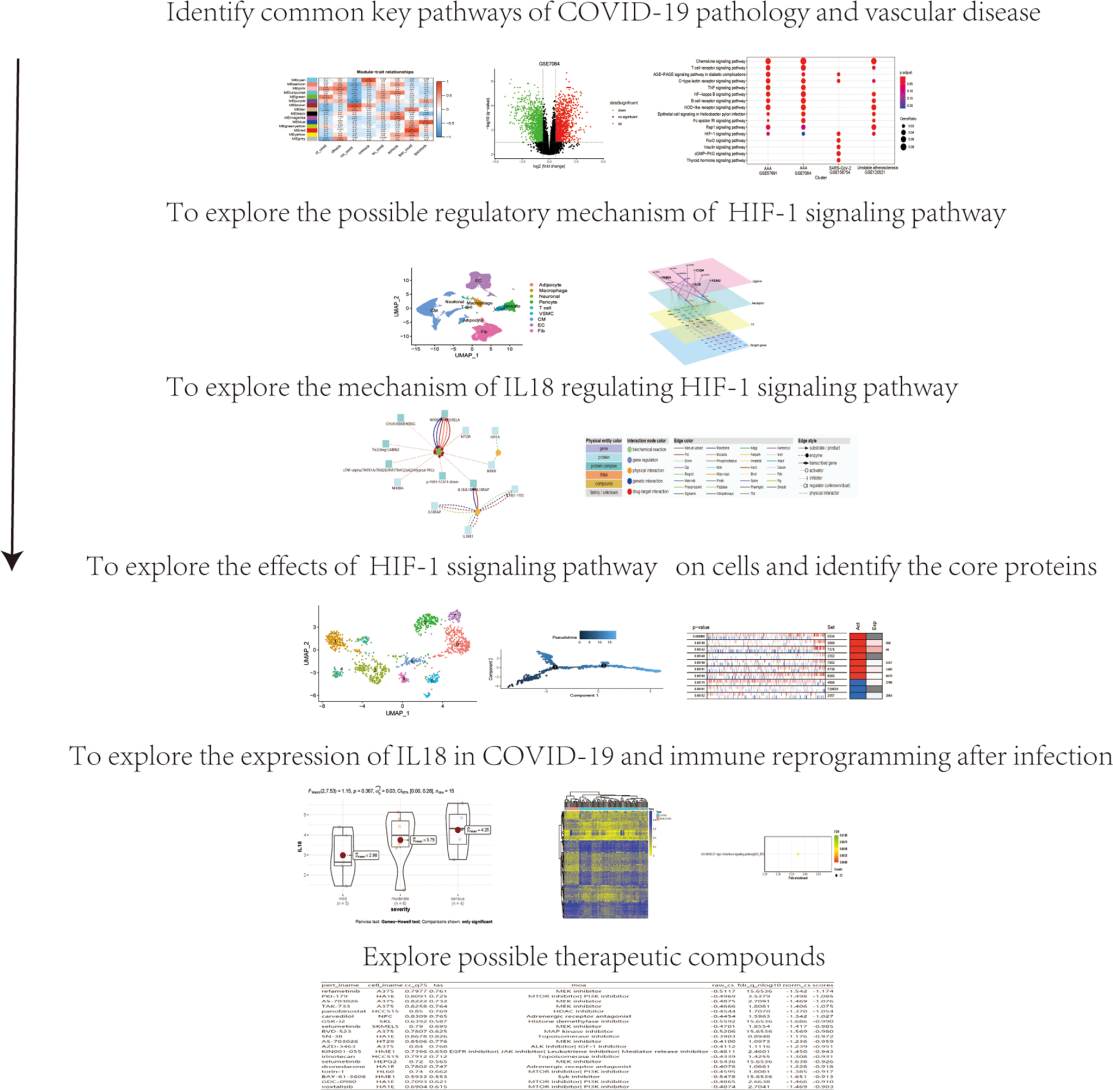
Data processing flow chart of this study.

As shown in **Figure 1**, data processing in this study was divided into 6 steps.

### The HIF-1 Signaling Pathway Is a Key Pathway for SARS-CoV-2 Infection, Aneurysms and Atherosclerosis

**Figure 2:**
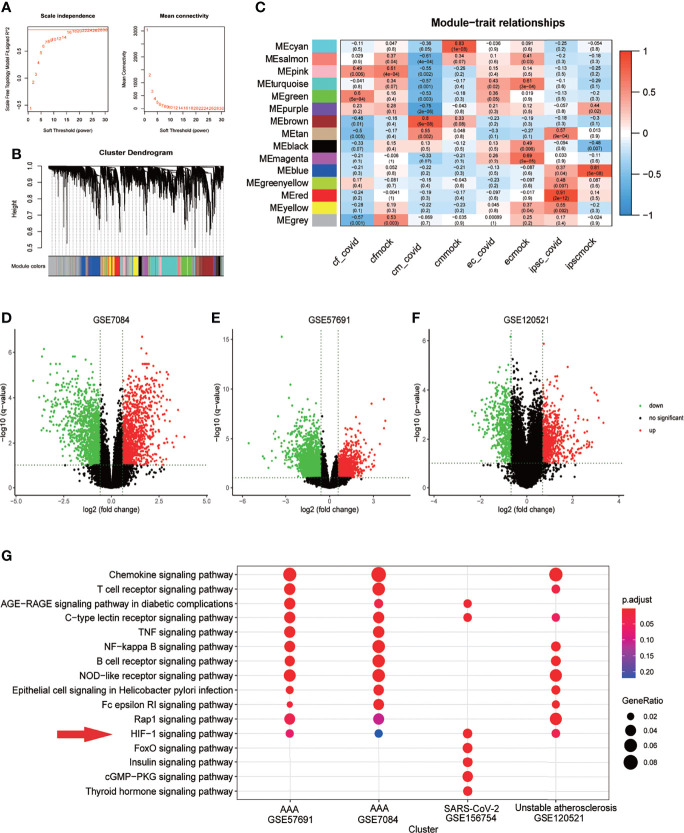
HIF-1 signaling pathway is a key pathway for SARS-COV-2 infection, aneurysm and atherosclerosis. **(A)** Soft threshold Screening for segmentation of inter gene regulatory network (Power =18) **(B)** Cluster dendrogram of genes in each module, gray module indicates that the genes have not been assigned to a specific module. **(C)** Correlation between each module and experimental group, red is positive correlation. The positive correlation module independent of simulated infection is considered as the core module of the corresponding group(cor>0 &Pvalue<0.05). **(D)**. Volcano plot of differentially expressed genes in GSE7084 (AAA versus normal, Limma, |logFC|>0.5 & q-value<0.1). **(E)**. Volcano plot of differentially expressed genes in GSE57691 (AAA versus normal, Limma, |logFC|>0.5 & q-value<0.1). **(F)**. Volcano plot of differentially expressed genes in GSE120521 (Unstable plaque versus stable plaque, Limma, |logFC|>0.5 & p-value<0.1). **(G)**. Comparison of KEGG enrichment results of differential genes from GSE7084, GSE57691, GSE120521 and GSE156754 (pvalue<0.05). HIF-1 signaling pathway was considered as the key pathway based on signal transduction pathways collected in KEGG database, which was marked with red snips.

In GSE156754, human induced pluripotent stem cells (iPSCs) and iPSCs-derived cardiomyocytes, cardiac fibroblasts, and endothelial cells were infected with SARS-CoV-2 and compared with uninfected cells. We performed WGCNA on this dataset and identified 15 regulatory modules with a soft threshold of 18, R square reaches 0.9. At this point, the network conforms to the scale-free distribution. (**Figure 2A**). Cluster dendrogram showed that gene expression in each module was highly correlated (**Figure 2B**). By mapping to the experimental treatment group, we identified five modules that were highly correlated with virus infection (green, brown, tan, greenyellow, red), which were believed to be the key genes after SARS-CoV-2 infection (Correlation coefficient>0 & P<0.05) (**Figure 2C**). The scatterplot showed that the gene expression pattern in the module had a good correlation with the corresponding groups (P<0.01) (**Supplementary Figure 1**). The volcano plot showed the differential genes of abdominal aortic aneurysm versus normal artery(GSE7084 and GSE57691) and atherosclerotic unstable plaque compared with stable plaque(GSE120521) (**Figures 2D–F**). KEGG enrichment analysis was performed for the key genes obtained from GSE7084, GSE57691, GSE120521 and GSE156754. Comparison of enrichment results revealed different signaling patterns between SARS-CoV-2 infection and aneurysm and atherosclerosis. After screening based on KEGG database Signal Transduction, HIF-1 signaling pathway was considered to be the common key pathway for SARS-CoV-2 infection, aneurysm and atherosclerosis (P.adjust<0.1 Benjamini & Hochberg) (**Figure 2G**).

### Macrophage-Derived IL18 Regulates HIF-1 Signaling Pathway Through IL18R1

**Figure 3:**
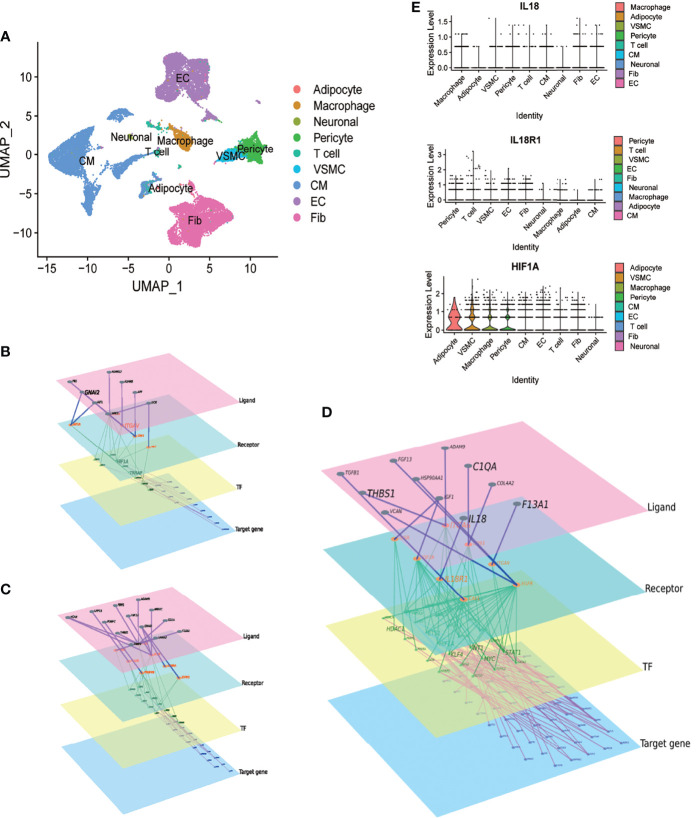
Macrophage-derived IL18 regulates HIF-1 signaling through IL18R1. A.UMAP scatter plot of cell types in the heart of COVID-19 patients derived from GSE171668. **(A)** total of 29,472 cells were included in 9 types. **(B)** The molecular regulatory network between macrophages and cardiomyocytes. **(C)** The molecular regulatory network between macrophages and vascular smooth muscle cells. **(D)** The molecular regulatory network between macrophages and endothelial cells. There are four layers of ligand, receptor, TF and Target genes in the networks, which were marked by different colors. Up-regulated genes in aneurysm and atherosclerosis were marked in **(B–D)**. **(E)** Expression distribution of IL18, IL18R1 and HIF-1A in different cell types.

In GSE171668, 29472 cells were grouped into 9 cell subtypes after dimensionality reduction. Cell annotation follows the annotation result of the original article, but the subclusters of each type were not specifically distinguished (**Figure 3A**). We explored the intercellular communications between macrophages and cardiomyocytes, VSMCs and endothelial cells, respectively. (**Figures 3B–D**). Combined with the differential genes obtained in aneurysms and atherosclerosis, the signaling molecules secreted by macrophages were further screened. The IL18/IL18R1/HIF1A regulatory relationship was obtained (**Figure 3D**). The expression distribution of IL18 IL18R1 and HIF1A in different cell types was sorted according to the expression. The expression of IL18 was highest in macrophages, while IL18R1 and HIF1A were widely distributed in vascular smooth muscle cells, macrophages, cardiomyocytes. These results suggest that IL18/IL18R1/HIF-1 signaling axis is widely applicable in the cardiovascular system (**Figure 3E**).

### NF-κB Mediates the Regulation of IL18/IL18R1 on HIF-1 Signaling Pathway

**Figure 4:**
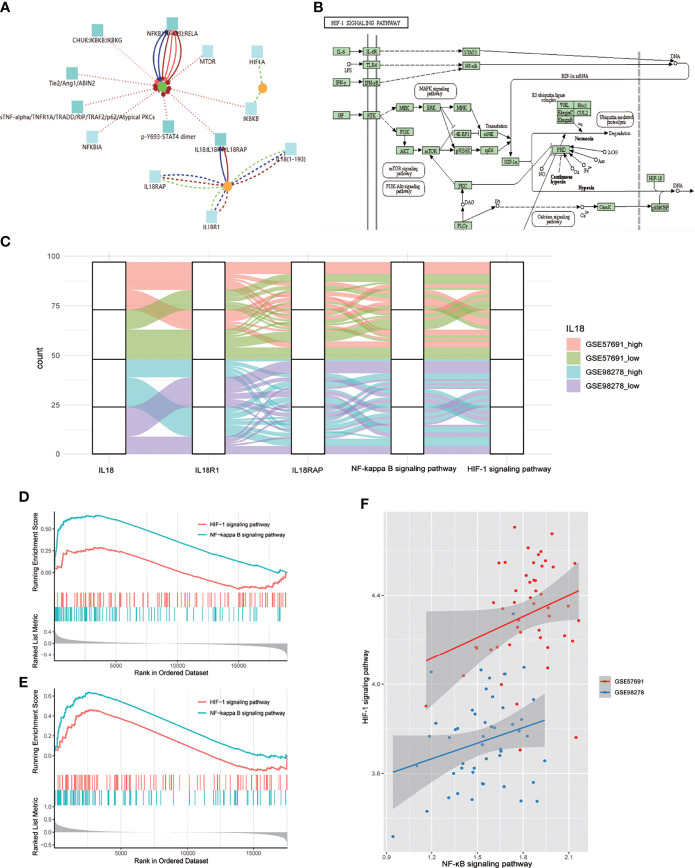
NF-κB mediates the regulation of IL18 on HIF-1 signaling pathway. **(A)** Proteomics analysis of IL18/IL18R1 and HIF-1A based on CPDB. IL18/IL18R1 can promote nuclear entry of NF-κB. **(B)** After entering the nucleus, NF-κB promotes HIF-1A transcription and activates HIF-1 signaling pathway. (KEGG: hsa04066) **(C)** Sankey diagram of IL18,IL18R1,IL18RAP,NF-κB and HIF-1 signaling pathways in GSE57691 and GSE98278. **(D)**. GSEA based on IL18R1 expression (GSE57691). **(E)** GSEA based on IL18R1 expression (GSE98278). **(F)** Scatter plot of correlation between NF-κB and HIF-1 signaling pathway expression scores. The scores were calculated by GSVA(GSE57691 and GSE98278).

CPDB-based analysis (http://cpdb.molgen.mpg.de/CPDB) showed that IL18/IL18R1 can promote NF-κB entry into the nucleus and activate related pathways through phosphorylation(**Figure 4A**). KEGG pathway map showed that NF-κB can promote HIF-1A transcription and participate in HIF-1 signaling pathway activation after entering the nucleus (**Figure 4B**). We evaluated the activation level of NF-κB and HIF-1 signaling pathway in aneurysm samples by GSVA. The samples in GSE57691 and GSE98278 were grouped and mapped according to IL18 expression, NF-κB and HIF-1 signaling pathway scores (**Figure 4C**). In GSE57691 and GSE98278, GSEA based on IL18 expression showed increased activation of NF-κB and HIF-1 signaling pathway after increased IL18 expression (**Figures 4D, E** **)**. Correlation analysis based on GSVA scores showed that NF-κB was positively correlated with the activation level of HIF-1 signaling pathway (GSE57691:nCor=0.27&P=0.05, GSE98278: Cor=0.26&P=0.07) (**Figures 4F**).

### Activated MAP2K1 and MAPK1 Suggest That MEK Pathway Is Involved in the Regulation of HIF-1 Signaling Pathway on Cell Phenotypes

**Figure 5:**
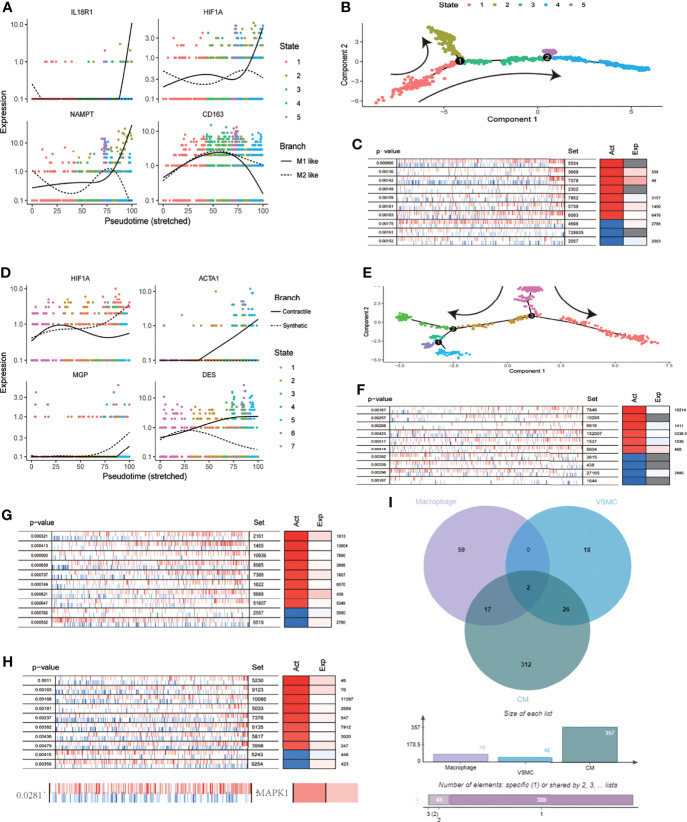
MEK signaling pathway was involved in the regulation of HIF-1A on cell function. **(A)** Expression distribution of IL18R1,HIF1A,CD163 and NAMPT on pseudo time locus in macrophages. The solid line represents M1 polarization direction, and the dotted line represents M2 polarization direction. **(B)** Pseudo-time locus analysis of polarization of macrophages **(C)** Top 10 proteins activation states derived from VIPER algorithm based on HIF-1 signaling pathway scores in macrophages. **(D)** Expression distribution of HIF1A, ACTA1, MGP and DES on pseudo time locus in VSMC. The solid line represents contractile direction, and the dotted line represents synthetic direction. **(E)** Pseudo-time locus analysis of phenotypic transformation in VSMC. **(F)** Top 10 proteins activation states derived from VIPER algorithm based on HIF-1 signaling pathway scores in VSMC. **(G)** Top 10 proteins activation states derived from VIPER algorithm based on HIF-1 signaling pathway scores in CM. **(H)** Top 10 proteins and MAPK1 activation states derived from VIPER algorithm based on HIF-1 signaling pathway scores in EC. **(I)** Venn diagram of activated proteins based on VIPER algorithm (macrophages, VSMC and CM). The two proteins obtained from intersection were MAP2K1 and EEF1A1.

Based on scRNA-seq data from the cardiovascular system after SARS-CoV-2 infection, we identified 1207 macrophages. Clustering dimension reduction showed heterogeneity of macrophages (**Supplementary Figure 2A**). Based on the clustering results, the genes with high variation in macrophages were obtained (**Supplementary Figure 2B**). Both IL18R1 and HIF-1A regulated the polarization direction of macrophages in the same way, leading to M1 polarization (**Figure 5A**). We used pseudo time trajectory analysis and combined with M1 and M2 macrophage markers to determine the direction of polarization. The expression levels of IL18R1, IL18RAP, HIF-1A, TGFBI, CD163 and NAMPT were mapped to the polarization trajectory. The solid line represented M1 polarization direction, and the dotted line represented M2 polarization direction. The direction of differentiation was marked with arrows in the diagram (**Supplementary Figure 2C** and **Figure 5B**). 78 proteins activated by HIF-1 signaling pathway were identified by VIPER analysis. The top 10 activating proteins sorted by p value were shown (**Figure 5C**). Consistent with the cell polarization trajectory, the expression of HIF-1 signaling pathway was accompanied by activation of CD86, which was a marker of M1 macrophages. The 737 VSMCs were divided into six clusters (**Supplementary Figure 2D**). The genes with high variation in SMCs were obtained through the characteristic genes of each cluster, which were used for the subsequent analysis of cell differentiation trajectory (**Supplementary Figure 2E** and **Figure 5E**). The expression levels of HIF-1A, ACTA1, MGP and DES were mapped to the differentiation trajectory. The solid line represented contractile phenotype direction, and the dotted line represented synthetic phenotype direction. The results showed that HIF1A promoted the synthetic phenotypic transformation of VSMCs (**Figure 5D**). Based on VIPER analysis, we identified 48 proteins which were activated with HIF-1 signaling pathway, the top ten were displayed (**Figure 5F**). Based on VIPER analysis, 357 proteins were identified in CM associated with HIF-1 signaling pathway, the top ten were displayed (**Figure 5F**). 146 proteins were identified in EC associated with HIF-1 signaling pathway. (**Figure 5G**). Venn diagram showed that MAP2K1 and EEF1A1 were common activated proteins in phenotypic transformation of macrophages, SMCs and cardiomyocytes (**Figure 5I**). In the results of VIPER analysis in endothelial cells, the P value of MAP2K1 was 0.1 and the NES was 1.46, the MAPK1’s P value and NES were 0.02 and 2.37 respectively. However, EEF1A1 showed no significant difference. Activated MAP2K1 and MAPK1 suggested that MEK pathway was involved in the regulation of HIF-1 signaling pathway on cell phenotypes transformation.

### The Expression of IL18 Was Positively Correlated With the Severity of COVID-19, and Decreased After Treatment, Epigenetic Changes of PBMC Led to the Activation of Type I Interferon Signaling Pathway

**Figure 6:**
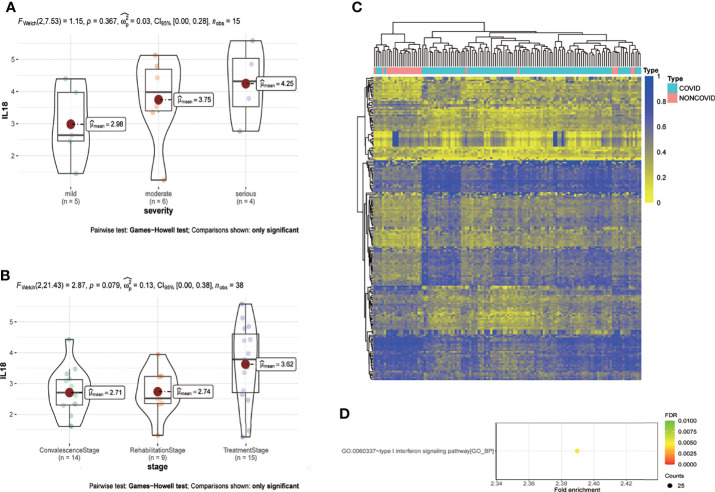
IL18 is involved in the progression of COVID-19, and the methylation pattern of PBMC changes after infection. **(A)** Expression of IL18 in patients with different clinical severity of COVID-19(mild:5 patients; moderate:6 patients; serious: 4 patients; p=0.367; Games-Howell test) **(B)** Expression of IL18 in patients with different stage of COVID-19(convalescence:14 patients; rehabilitation:9 patients; treatment: 15 patients; p=0.079; Games-Howell test) **(C)** Heat map of PBMC methylation differential sites after SARS-CoV-2 infection **(D)** Enrichment analysis of differential methylation sites after SARS-CoV-2 infection. (Pvalue<0.05).

Analysis based on GSE157859 data set showed that the expression of IL18 was positively correlated with the severity of COVID-19 (**Figure 6A**), and the level of IL18 decreased after treatment (**Figure 6B**). To study the epigenetic changes of PBMC after COVID-19, GSE173568 was analyzed. The results showed that the DNA methylation pattern of PBMC was significantly changed due to immune memory after SARS-CoV-2 infection (**Figure 6C**), but the epigenetic modification of IL18 and its possible transcription factors were not significantly changed. However, the enrichment analysis of differential methylation sites showed that the only signal pathway, namely type I interferon signaling pathway, with a confidence threshold of 0.05 (**Figure 6D**). Previous studies have shown that type I interferon is essential for IL18 expression, increased type I interferon expression promotes IL18 expression (17); macrophages that lack type I IFN signal were impaired in promoting IL-18 induction (18), chronic interferon transmission can lead to cytokine imbalance, including IL18 (19).

### MEK Inhibitors Are Promising as a Treatment for HIF-1 Signaling Pathway Overactivation

**Figure 7:**
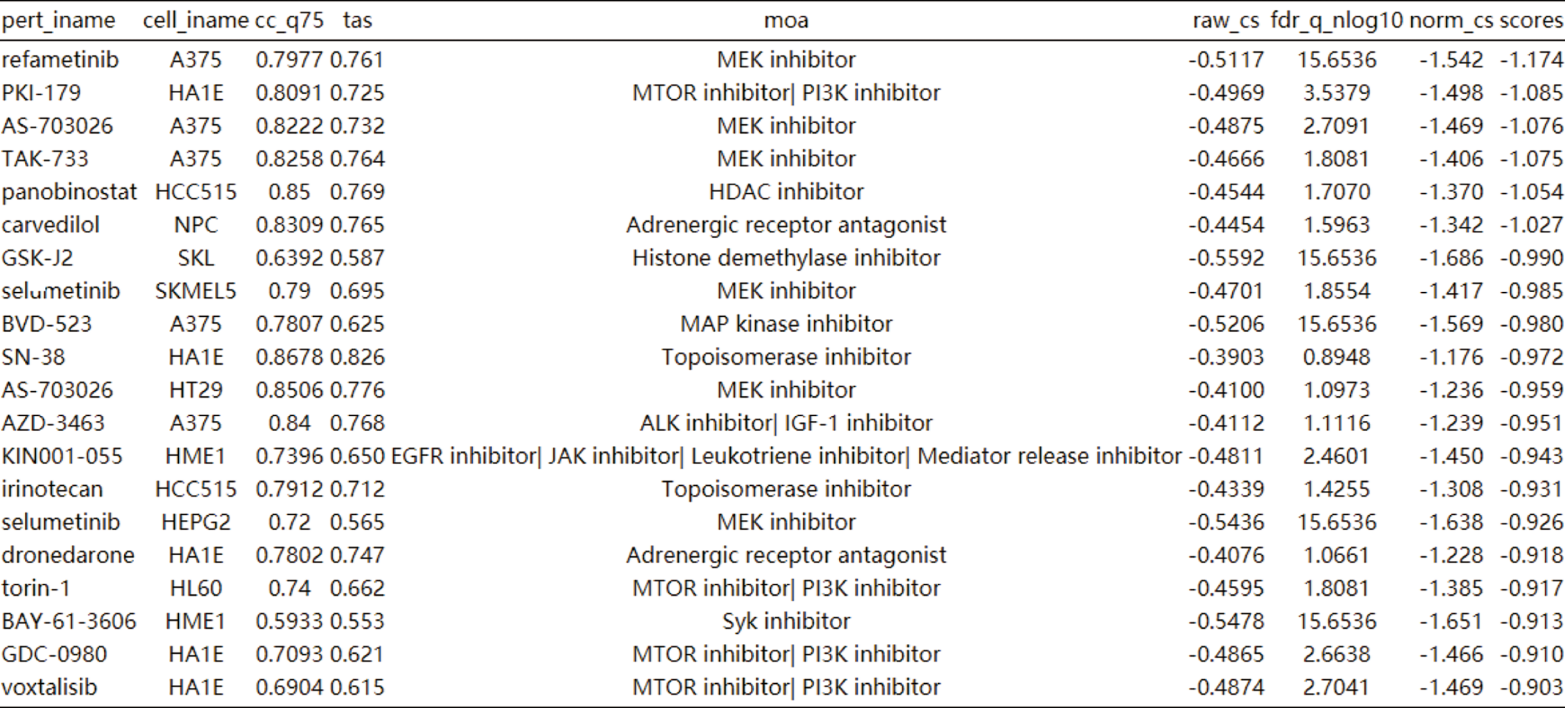
Cmap analysis after activation of HIF-1 signaling pathway. The first 20 compounds are shown.

CLUE TOUCHSTONE analysis tools screen for potential therapeutic compounds by comparing their effects on the cell transcriptome with the targeted differential gene set. Compounds were screened by transcriptome activity score and similarity score. Among the regulatory relationships of compounds on cells, a total of 12,143 regulatory relationships had a similarity score of less than 0 with HIF-1 signaling pathway. 3404 compounds involved in 1001 regulatory mechanisms were identified as having potential therapeutic value. The first 20 compounds after sorting include six MEK inhibitors and four mTOR inhibitors **(** **Figure 7** **)**. Adrenergic inhibitors may also have therapeutic effects, previous transcriptional molecular results showed that the estrogen signaling pathway was suppressed, and estrogen receptor activators had therapeutic potential. This may explain the better prognosis of women after SARA-CoV-2 infection.

In the VIPER analysis of this study, we found that MEK signaling pathway played a key role in the regulation of HIF-1 signaling pathway on cell phenotypes, which was consistent with the predicted results of therapeutic compounds. Thus, we identified the therapeutic effect of MEK inhibitors on cardiovascular risk after SARA-CoV-2 infection.

## Discussion

COVID-19 has become a serious threat to global health, resulting in tens of millions of infections and millions of deaths. At present, all countries in the world are trying to achieve herd immunity against SARS-CoV-2 by means of universal vaccination. Anti-inflammatory, anti-infective or anti-cytokine storm measures are commonly used in clinic. However, no specific target for post-viral infection has been developed. Symptoms of COVID-19 patients are heterogeneous, ranging from asymptomatic infection to ARDS and multiple organ failure, including the cardiovascular system, are observed in patients; but the mechanism is unclear. In addition, the risk of vascular diseases such as aneurysms and atherosclerosis after COVID-19 has not been clearly discussed.

In this study, we studied SARS-CoV-2 infection of endothelial cells, cardiomyocytes and fibroblasts induced by pluripotent stem cells through WGCNA. HIF-1 signaling is considered to be a key pathway in viral infection of the cardiovascular system. Previous studies have also found that HIF-1 signaling pathway is abnormally activated in PBMC of patients after viral infection, and its expression is higher in elderly patients, which may be related to the activation of HIF-1 pathway induced by SARS-CoV-2 ORF3a (9). It is noteworthy that HIF-1α also extensively promotes infection of other viruses. HIF-1A is expressed in various cells of the cardiovascular system to varying degrees, which significantly affects cell function and plays an important role in many cardiovascular diseases. Our study confirmed the critical role of HIF-1 signaling in aneurysm and atherosclerosis, HIF-1α signaling pathway was involved in the formation and rupture of AS plaques (20). When cells are hypoxic, HIF-1α enhances the transcription and expression of VEGF, thereby stimulating new angiogenesis, promoting AS and increasing plaque instability, promoting plaque rupture and inducing acute cardiovascular events, which may be the pathogenesis of acute coronary syndrome (21). Currently, the correlation between HIF-1 signaling pathway and hypertension has been observed, but the mechanism is unclear. PHD2 may regulate HIF-1 regulation of blood pressure (22), under hypoxia, HIF-1α can activate and increase the expression of iNOS and ET-1, and thus cause pulmonary vasoconstriction, which may be the mechanism of pulmonary hypertension. In this study, we identified the involvement of HIF-1 signaling pathway in abdominal aortic aneurysms. Previous research showed that HIF-1 signaling pathway is involved in the formation of arterial dissection (23). Therefore, we suggest that abnormal activation of the HIF-1 signaling pathway caused by SARS-CoV-2 infection is a possible mechanism for increased the risk of peripheral vascular disease, such as aneurysms and atherosclerosis.

In this study, the vascular smooth muscle cells (VSMCs) and endothelial cells (ECs) annotated in the scRNA-seq data derived from the coronary system and were consistent with the compositions of the peripheral blood vessel walls, so as the pathological changes. In addition to macrophage infiltration (24, 25), phenotypic transformation of VSMCs is a key mechanism in aneurysms (26) and atherosclerosis (27), so as the endothelial dysfunction (28, 29). It is noteworthy that macrophages have regulatory effects on vascular smooth muscle cells (30), endothelial cells and macrophages themselves (31). Meanwhile, macrophage infiltration was a pathological feature of the cardiovascular system after SARS-CoV-2 infection (32). Therefore, in order to study the regulatory mechanism of HIF-1 signaling pathway in the cardiovascular system microenvironment, macrophages were used as hub cells to conduct intercellular communication analysis, and then the IL18/IL18R1/HIF-1 signaling pathway axis was obtained. IL18 is a pro-inflammatory cytokine in the IL-1 family expressed by macrophages. Processed into an active form by Caspase-1, which stimulates interferon γ production and modulates the helper T (Th) 1 and Th2 responses (33). This cytokine has been associated with damage to different organs and potentially fatal conditions characterized by cytokine storms. It is mainly involved in polarization-assisted 1 (Th1) cell and natural killer cell (NK) immune responses. Our study shows that IL18 also promotes M1 polarization of macrophages. Il-1 β and IL-18 inhibition have been shown to be effective in reducing major cardiac adverse events in clinical trials, including myocardial infarction and heart failure (34). IL18 has also been reported to be involved in abdominal aortic aneurysm formation and atherosclerosis (35, 36). Our study suggests that IL18 is involved in the development of cardiovascular diseases through HIF-1 signaling pathway. Previous studies have shown that NFκB mediates the regulation of HIF1A by IL18RAP (37). Our study showed that IL18 receptor IL18R1 was also involved in HIF-1 signaling *via* NFκB.

We found significant changes in epigenetic modification patterns of PBMC after SARS-CoV-2 infection, which may be related to the reprogramming of immune memory. By analyzing the differential methylation sites, we obtained the only signal pathway, namely the type I interferon signaling pathway. The type I interferon response is critical for providing effective protection against viral infection and is triggered rapidly by host sensors (e.g., viral nucleic acids) that recognize pathogen-associated molecular patterns (PAMPs). Activation quickly induces the expression of hundreds of genes called interferon-stimulating genes (ISGs), which in turn control other downstream molecules, including pro-inflammatory cytokines (38); these cytokines participate in direct inhibition of virus replication, recruitment and activation of various immune cells. IFN-I promotes viral clearance and tissue repair, and induces a long-term adaptive immune response to the virus. Type I IFN signaling is critical for IL-18 induction (39), and macrophages lacking type I IFN signaling are impaired in their ability to promote IL-18 induction (18).

In this study, we found that MEK and mTOR inhibitors have potential therapeutic value in overactivation of HIF-1 signaling pathway. Especially MEK inhibitors, which were consistent with the results of VIPER analysis. Ras/Raf/MEK/ERK and PI3K/Akt/mTOR signaling pathways are responsible for regulating cell growth, proliferation, survival and apoptosis; and have been considered as promising targets for cancer therapy (40). Connections between extracellular signals and cytoplasmic and nuclear effectors, various growth factors, adhesion molecules and differentiation factors utilize Ras/Raf/MAPK/extracellular signal-regulated kinase (Ras/Raf/MEK/ERK) pathways (41). Ras/Raf/MEK/ERK mediates different signaling pathways, including activation of JAK2/STAT3, PI3K/Akt, and activation of IGF1, VEGF, and SDF-1A signaling cascades of ERK and PI3K/Akt. Rapamycin (mTOR) targets a variety of biological processes including protein synthesis, cell growth, proliferation, autophagy, lysosomal function and cell metabolism (42). In the cardiovascular system, the mTOR pathway regulates physiological and pathological processes of the heart and is essential for fetal cardiovascular development and postnatal cardiac homeostasis. Studies have shown that mTORC1 activation is essential for the development of adaptive cardiac hypertrophy, and partial genetic or pharmacological inhibition of mTORC1 can reduce cardiac remodeling and heart failure. In addition, mTORC1 inhibitors reduce cardiac dysfunction caused by genetic and metabolic disorders (43). Our previous study showed that rapamycin, an mTOR inhibitor, can inhibit intimal hyperplasia (44). Current studies showed that both ERK (45, 46) and mTOR signaling pathways (47, 48) can activate HIF-1 signaling pathway.

Interestingly, we found that estrogen receptor activators antagonize transcriptomic changes resulting from overactivation of the HIF-1 signaling pathway. This is consistent with clinical observations of better outcomes in women with SARS-CoV-2 infection (5). In fact, the interaction between estrogen and the HIF1 signaling pathway has been demonstrated in other studies (49). A possible explanation is that ER competes with HIF1 in intracellular ubiquitination mediated degradation. Studies showed that ERα, HIF1α, HIF2α are the target substrates of pVHL in cells, which are ubiquitinated and then degraded. When HIF1A is overactivated, ERα binds to pVHL and is degraded by ubiquitination, thus inhibiting estrogen signaling (50).

## Conclusion

In this study, we found that macrophages increase cardiovascular risk through the IL18/IL18R1/HIF-1/MEK signaling pathway after SARS-COV-2 infection. MEK inhibitors may be an option for cardiovascular protection after SARS-COV-2 infection.

## Data Availability Statement

The original contributions presented in the study are included in the article/**Supplementary Material**, further inquiries can be directed to the corresponding author.

## Author Contributions

LZ, and HB, conceptualization. LZ, methodology, software, formal analysis, and original draft preparation. ZW, ML, and PS, validation. SW and CZ, investigation and resources. HW, data curation. PS and SW, review and editing. LZ and PS, visualization. ML, supervision. HB, project administration and funding acquisition. All authors contributed to the article and approved the submitted version.

## Funding

This study was funded by the National Natural Science Foundation of China to Hualong Bai (Grant No: 81870369) and Key Projects of Medical Science and Technology in Henan Province (Grant No: SBGJ202002035).

## Conflict of Interest

The authors declare that the research was conducted in the absence of any commercial or financial relationships that could be construed as a potential conflict of interest.

## Publisher’s Note

All claims expressed in this article are solely those of the authors and do not necessarily represent those of their affiliated organizations, or those of the publisher, the editors and the reviewers. Any product that may be evaluated in this article, or claim that may be made by its manufacturer, is not guaranteed or endorsed by the publisher.
