# Supplementary figures and images for: Cardiovascular Risk After SARS-CoV-2 Infection Is Mediated by IL18/IL18R1/HIF-1 Signaling Pathway Axis
Source: Front Immunol. 2022 Jan 5;12:780804. doi: 10.3389/fimmu.2021.780804 (PMC8766743; doi:10.3389/fimmu.2021.780804)

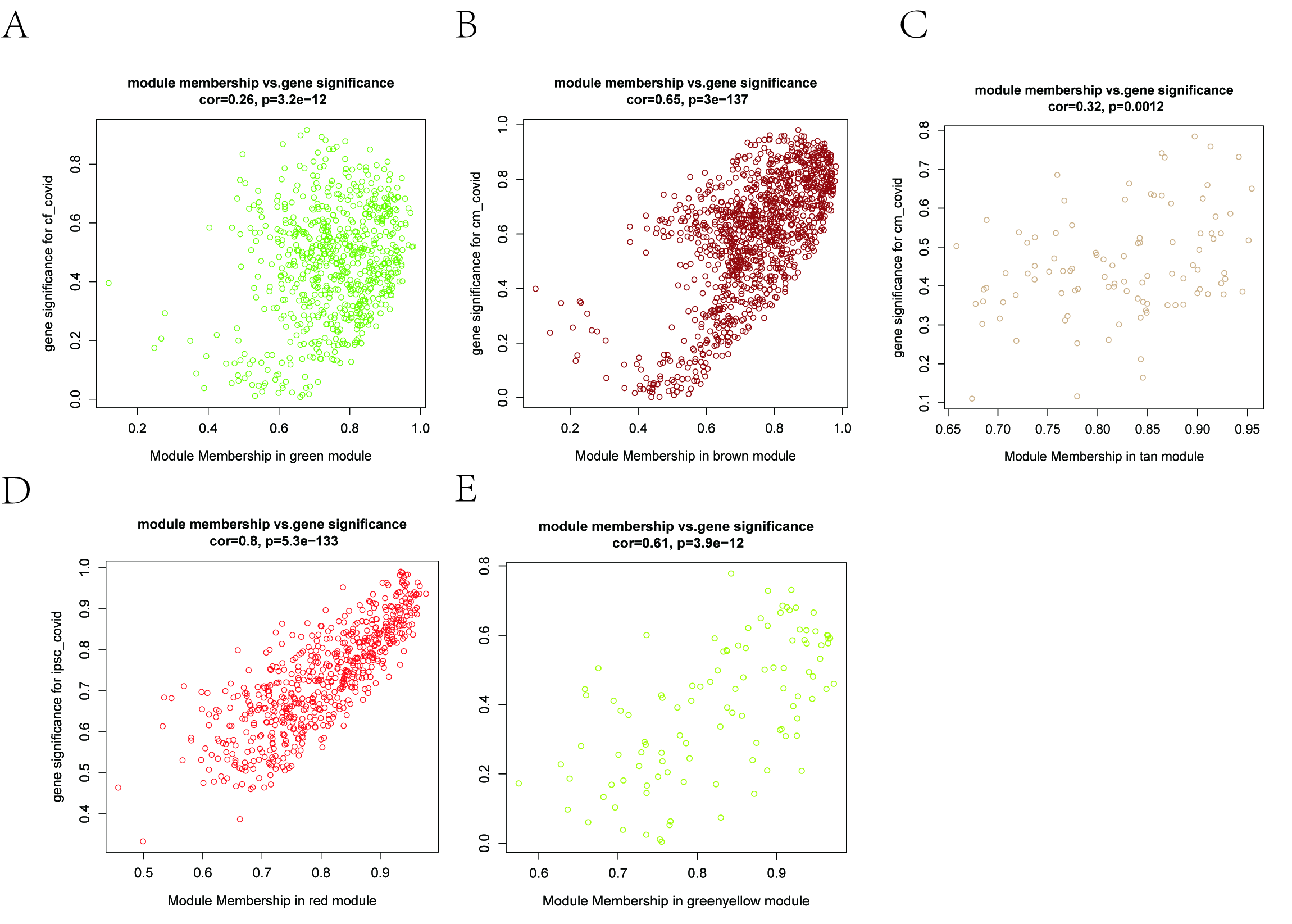

Supplement: Supplementary file 1 [file Image_1.tif]

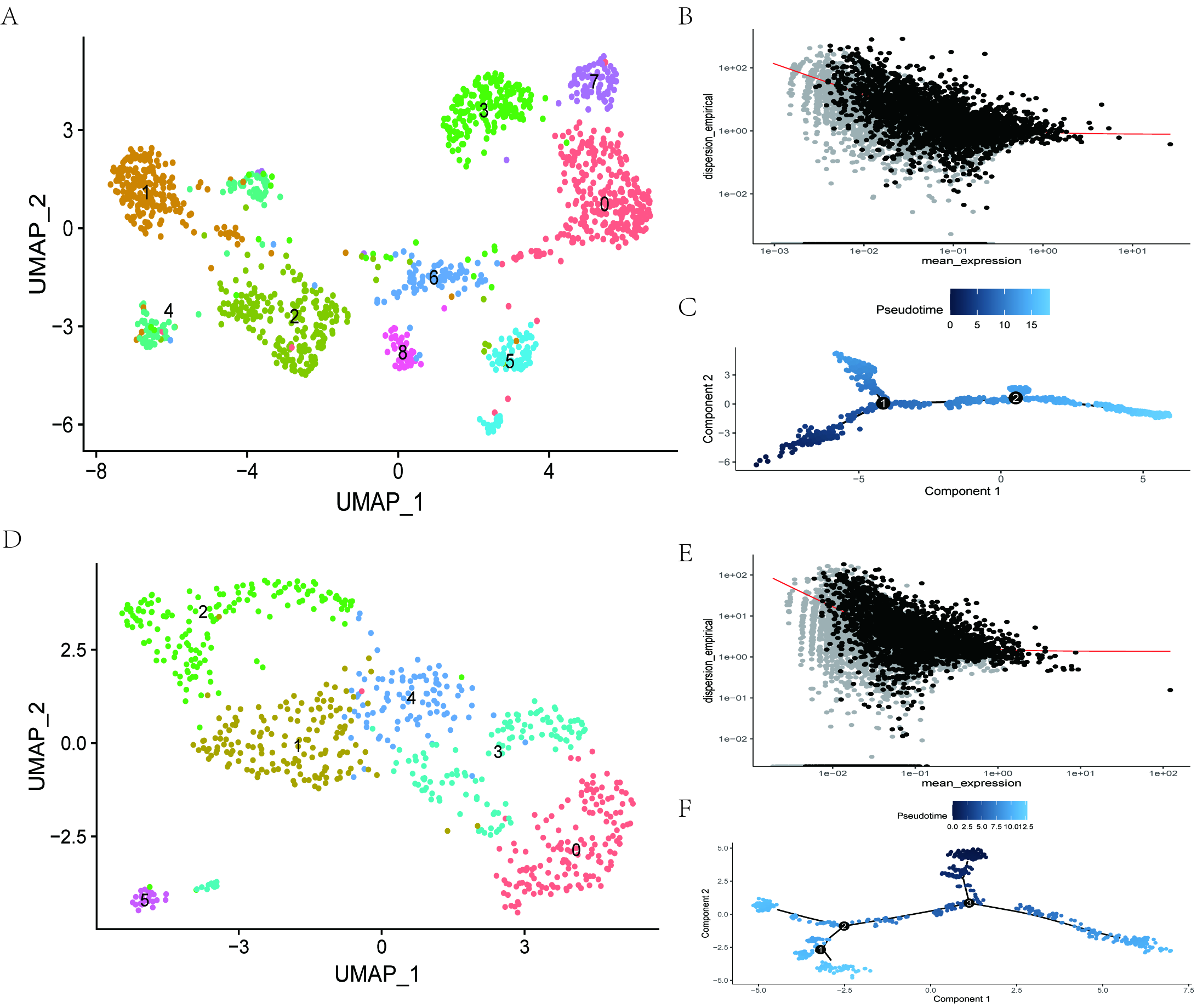

Supplement: Supplementary file 2 [file Image_2.tif]
